# Age-Related Diseases and Clinical and Public Health Implications for the 85 Years Old and Over Population

**DOI:** 10.3389/fpubh.2017.00335

**Published:** 2017-12-11

**Authors:** Efraim Jaul, Jeremy Barron

**Affiliations:** ^1^Skilled Nursing Department, Herzog Hospital, Jerusalem, Israel; ^2^Hebrew University of Jerusalem, Jerusalem, Israel; ^3^Johns Hopkins University, Baltimore, MD, United States; ^4^Herzog Hospital, Jerusalem, Israel

**Keywords:** oldest-old, medical decision-making, public policy, aging, longevity

## Abstract

By 2050, the American 85 years old and over population will triple. Clinicians and the public health community need to develop a culture of sensitivity to the needs of this population and its subgroups. Sensory changes, cognitive changes, and weakness may be subtle or may be severe in the heterogeneous population of people over age 85. Falls, cardiovascular disease, and difficulty with activities of daily living are common but not universal. This paper reviews relevant changes of normal aging, diseases, and syndromes common in people over age 85, cognitive and psychological changes, social and environmental changes, and then reviews common discussions which clinicians routinely have with these patients and their families. Some hearing and vision loss are a part of normal aging as is decline in immune function. Cardiovascular disease and osteoporosis and dementia are common chronic conditions at age 85. Osteoarthritis, diabetes, and related mobility disability will increase in prevalence as the population ages and becomes more overweight. These population changes have considerable public health importance. Caregiver support, services in the home, assistive technologies, and promotion of home exercise programs as well as consideration of transportation and housing policies are recommended. For clinicians, judicious prescribing and ordering of tests includes a consideration of life expectancy, lag time to benefit, and patient goals. Furthermore, healthy behaviors starting in early childhood can optimize quality of life among the oldest-old.

## Background

The percentage of national populations over age 65 has been increasing in the last 10 years and will continue to rise for another 20 years due to improved life expectancies and a post-World War 2 baby boom. Beginning in 2030, the numbers of adults over age 85 will rise quickly. By 2050, the number of adults over age 80 around the globe will triple from 2015 numbers (1). Some nations are aging even faster. Now is the time for the public health community to plan for the “older-older age wave.” Many cities have begun to explore how to make themselves more “elder-friendly.” As the baby boom-generation ages from 65 to 85, there will be a more intense need for services in the home and in community and institutional settings.

The aging process currently encompasses more than a generation and exceeds three decades. The common framework for describing different older adult populations is “young-old” (2), “old” (3), and “old-old.” The “young-old” are people in their 60s and early 70s who are active and healthy. The “old” are people in their 70s and 80s who have chronic illnesses and are slowing down with some bothersome symptoms. The “old-old” or “oldest-old” (4) are often sick, disabled, and perhaps even nearing death.

When caring for older adults as a clinician or as a caregiver, predicting the future and then planning for the most likely aging trajectories are key steps. This paper presents a model for the clinical and public health needs of adults over age 85.

The changes associated with a chronologic age of 85 can be divided into a few domains: normal aging, common diseases, and functional, cognitive/psychiatric, and social changes.

## Normal Aging

Although changes can be described in every organ system, this review will address changes with public health and clinical decision-making implications.

### Sensory Changes

#### Hearing Loss

Hearing loss (presbycusis) and increased cerumen production with aging contribute to difficulty hearing. The prevalence of hearing loss increases as a function of age and accumulating risk factors and has a high association with reduced quality of life (5). Approximately one-half of adults over age 85 have hearing impairment (6). Mild hearing loss can impair speech processing, particularly if speech is rapid or if multiple talkers in large rooms generate reverberant noise. Therefore, verbal communication difficulties are most prominent in settings where people gather. Increased social isolation mediates the observed associations between hearing loss and depression, cognitive decline, and reduced quality of life.

The use of hearing aids could reverse adverse effects on the quality of life, and cognitive function in elderly adults (7). Unfortunately, among individuals with hearing loss in one study, only 14.6% reported currently using a hearing aid (8). Often, health insurance does not offer coverage for these devices.

#### Visual Acuity

Visual acuity decreases normally with age (presbyopia). Older adults will often have problems with glare, making night driving riskier. A (strike in) longitudinal survey conducted in the UK on the population aged 75 and older found that prevalence of severe visual impairment was 23% at ages 85–89 and increased to 37% at age over 90 (9). Visual acuity deteriorates faster at higher ages. Cataract surgery is typically safe and sometimes helps function.

#### Vestibular Function

Dizziness is a common multifactorial geriatric syndrome contributing to falls. Vestibular function declines subtly with age. Vestibular rehabilitation can be an effective treatment (10).

### Muscle Strength and Fat Changes

Muscle mass and strength decline starting in the fourth decade of life. By age 85, approximately 20% of people meet criteria for sarcopenia (meaningful loss of muscle mass and strength) (11). Chronic inflammation, declining hormone levels, impaired muscle mitochondrial function, and impaired muscle stem cell function all probably contribute to sarcopenia (12). This decline in muscle mass and increase in fat mass contributes to important changes in pharmacokinetics. Older adults may need lower medication doses than younger adults. Muscle weakness (13) and rapid rate of strength decline (14) both predict future mortality.

### Immunosenescence

There are a wide variety of age-related changes in the immune system, some mediated by chronic inflammation and a chronic pro-inflammatory state. There is a decline in B cell function, a decline in T cell generation, altered T cell activation, and dysfunction of innate immunity (including impaired neutrophil function and chemotaxis and a dysregulated proinflammatory monocyte response). These changes (15) weaken the body’s capacity to fight infection. For example, influenza infections are more common and more serious in older adults while the vaccine is less effective. Cellular immune dysfunction also contributes to the prevalence of herpes zoster among older adults. Vaccines are generally not as effective for older adults. High doses of the influenza vaccine may be more helpful than standard doses (16). Chronically slowed inflammatory processes also contribute to slow wound healing in older adults (17).

### Urologic Changes

The urinary bladder is often not sterile in older adults but rather is colonized with bacteria not causing infection. Asymptomatic bacteriuria is more common in women than men and is most frequent among hospitalized patients and residents of long-term care facilities (up to 50% of women in these high risk groups) (18). Use of antibiotics in this situation is inappropriate (19) and may contribute to antimicrobial resistance.

## Somatic Disease and Multiple Chronic Conditions

### Cardiovascular Disease

Cardiovascular disease remains the most common cause of death of older adults, although death rates have dropped in the last 20 years. This category includes chronic ischemic heart disease, congestive heart failure, and arrhythmia. Ischemic heart disease may be underdiagnosed in the oldest-old (20). Normal aging includes vascular remodeling and vascular stiffness (21). Atherosclerosis causes inflammation and further vascular changes (22) increasing risk for cardiac events, cerebrovascular events, peripheral vascular disease, cognitive impairment, and other organ damage.

### Hypertension

Hypertension, a major contributor to atherosclerosis, is the most common chronic disease of older adults (23). Isolated systolic hypertension is particularly common among older adults and is associated with mortality even at advanced ages. The value of intensive pharmacotherapy for hypertension in people over age 75 remains controversial. Evidence seems to suggest that aggressive treatment should be offered (24) and continued as long as it is well-tolerated and consistent with the patient’s goals.

### Cancer

Cancer is the second leading cause of death in older adults. However, by age 85, the death rate from cancer begins to fall (25). Slow-growing tumors seem to be common in this population.

Response to cancer treatment depends on functional status rather than age. Individuals in their ninth or tenth decade should not be denied aggressive cancer treatment simply due to age.

Screening is not recommended for breast cancer after age 75, due to insufficient evidence for benefit, although there may be benefit for women with a long life expectancy (26, 27). Similarly, for people over age 75 in the US, colon cancer screening is only recommended in cases where there is a long predicted life expectancy and a perceived strong capacity to tolerate cancer treatment, if needed (27, 28). At any age, life expectancy is quite variable in older adults, based on comorbidities and other factors (29).

Screening for prostate cancer is not recommended due to frequent false positives, which are burdensome, and to identification of slow-growing tumors (30).

### Osteoarthritis

Osteoarthritis is the second most common chronic condition (23) among American older adults and a common cause of chronic pain and disability. Fifty-two percent of 85-year olds had a diagnosis of osteoarthritis in one study (20). The prevalence of osteoarthritis seems to be higher among women than men. Obesity is a risk factor for osteoarthritis and as the population ages (and particularly as the overweight population ages), the rate of severe hip, and knee arthritis will increase. Pain management will continue to be a vexing clinical and health policy problem as virtually all analgesics have remarkable risks in older adults. Osteoarthritis treatments also include costly joint replacement surgery, which is often accompanied by intensive rehabilitative therapies. Low back pain is itself a common symptom particularly in older women and the cause is often multifactorial. Non-pharmacologic treatments can help.

### Diabetes Mellitus

Diabetes rates have been increasing as populations age and become more overweight. The prevalence of diabetes among American older adults may increase more than 400% by 2050 (31). Diabetes remains a strong risk factor for cardiovascular disease at age 85 (32). Diabetes is also associated with peripheral arterial disease and peripheral neuropathy, contributing to diabetic foot ulcers and amputations. Diabetic foot ulcers occur in 6% of diabetic patients annually and amputations in about 0.5%. Management approaches in diabetes should be individualized. Sulfonylureas and insulin carry a substantial risk of hypoglycemia and use should be weighed carefully in vulnerable older adults. Transitions from hospital to home or post-acute care are risky times for patients treated with hypoglycemic agents as dosing needs may fluctuate (31). Regular foot examinations are critical for people with diabetes to prevent amputations. Regular walking can improve circulation in the legs.

### Osteoporosis

Osteopenia is normal loss of bone density with aging. Many 85-year-old adults have osteoporosis, a more severe weakening of bone density. Osteoporosis is associated with an increased rate of bone fractures, while osteopenia is not. Bone density screening is recommended for women over age 65 (33). Although the prevalence of fractures in men increases by age 85, the value of osteoporosis screening for men has not been clearly demonstrated. The effectiveness and safety of calcium and vitamin D supplementation in order to prevent fractures remains controversial.

### Multiple Chronic Conditions

Sixty two percent of Americans over 65 have more than one chronic condition (34) and the prevalence of multiple chronic conditions is increasing (35), due to aging of populations and to increasing diabetes rates. Older adults with multiple chronic conditions account for a large percentage of health spending (36). Targeting this population for research and for quality improvement should improve care and reduce costs.

## Physical Function

Normal age-related changes and accumulated pathology contribute to functional changes seen with aging.

### Walking Speed

Walking speed declines with normal aging but will decline additionally due to disease. Walking speed measurements can be used to predict future community ambulation, falls, disability (37), and risk of mortality (38). Measurement of walking speed is quick, safe, requires no special equipment, and adds no significant cost to clinical care. In one study, the average walking speed for the age group of 85–89 is 1.1 m/s for men and 0.8 m/s for women. After age 90 years, mean waking speed decreased to 0.9 m/s for men and 0.8 m/s for women (39). Physical activity interventions can improve walking speed.

### Mobility Disability

Seventy-three percent of Americans over age 85 have some difficulty with walking according to a US Census study. Mobility disability is associated with social isolation, falls, and depression. One-third of people over age 85 with a disability live alone (40).

### Disability in Activities of Daily Living

Disability rates are relatively high among adults over age 85. Rates of disability in activities like dressing and bathing, and disability in instrumental activities of daily living such as cooking, all rise with age over 80. Difficulty with bathing typically precedes difficulty with dressing or difficulty with using the toilet. In one study, 75% of people aged 85 had difficulty or disability with bathing and 25% had difficulty or disability with using the toilet (41). People with disabilities often also struggle with chronic pain, depression, and complex medication regimens (42). The percentage of older adults with disabilities has modestly decreased in recent decades.

### Falls

Falls are a major cause of morbidity and disability among older adults. 30–40% of adults over age 70 fall each year and rates are particularly high for older adults in long-term care facilities. Falls account for more than half of injuries among older adults. Fall-related death rates are higher for adults over age 85 than for other age groups (43). Physical activity, vitamin D supplementation, balance exercise, and home safety assessment as a part of a multifactorial fall prevention program have been shown to reduce the incidence of falls (44). Individuals with balance problems or falls should have a multifactorial falls risk assessment (45).

### Frailty

Frailty is defined as special vulnerability to stressors and is suggested by weakness, slowness, exhaustion, and weight loss (46). In one study, 38% of people aged 85–89 were frail (47). Frailty status can be assessed easily and the frail state predicts future disability, falls, hospitalizations, and poor surgical outcomes. Targeted interventions for frail populations would likely include physical activity and nutritional components (48) as well as medication reviews.

### Continence

Thirty percent of women over age 65 and 50% of older adults in nursing facilities have urinary incontinence (49). Common causes for incontinence among women include overactive bladder, stress incontinence, and functional incontinence. Urinary incontinence reduces well-being and quality of life (50). However, common incontinence medications cause burdensome side effects.

## Psychological and Cognitive

### Cognitive Aging

Mild short-term memory loss, word-finding difficulty, and slower processing speed are normal parts of aging that are often noticeable by age 85. Changes from normal brain aging can affect driving safety and increase risk for financial exploitation. These changes can also reduce capacity to understand complicated medical information. Brain aging does not happen at a uniform rate and genetic and social factors (like education and occupation) may be protective (51). Normal cognitive aging does not lead to dementia.

Not all brain functions decline with age. Wisdom and knowledge are known to increase with normal aging, contributing to the appropriate respect afforded to community elders. Empathy and altruism also may increase with age (52).

### Dementia

Rates of dementia increase with age. Death rates from Alzheimer’s disease have been rising while death rates for cardiovascular disease have been falling. Worldwide dementia prevalence may rise from 47 million in 2015 to 131 million in 2050. The estimated cost of dementia worldwide was $818 billion in 2015 and is expected to grow to $2 trillion by 2030 (53). Although dementia screening may have limited clinical benefit because medications are only marginally effective, screening may have public health benefit. Many older adults with dementia have unmet needs and may be living or driving unsafely (54). The Folstein Mini-Mental State Examination is the most commonly used instrument to diagnose dementia but has several limitations including an educational adjustment. For example, in the Irish Longitudinal Study on Aging, mean MMSE scores for 85-year olds with poor education was 25.2 while mean score for 85-year olds with good education was 28.0 (55). People with dementia need opportunities for cognitive stimulation, caregiver support, and possibly assistive technologies to improve safety and independence.

### Depression

Depression is not a normal consequence of aging. Grief can be a normal response to life events that occur with aging such as bereavement; retirement/loss of income; and loss of physical, social, or cognitive function from illness. Major depression is common throughout adulthood but incidence rates drop after age 60 and then rise again after age 80. Depression prevalence for adults over age 85 is double the rate seen at age 70–74 (23). Depression is even more common among institutionalized older adults and those with disabilities (56). Aggressive approaches to diagnosis and treatment are warranted to minimize suffering, improve overall functioning, and prevent suicide. Suicide among older American adults is most common in 85-year-old white men (57).

## Social/Environmental

Being married and being wealthy predict longer survival. The benefit of marriage seems stronger for men than women. Alternatively, social isolation predicts mortality and other adverse outcomes in older adults (58). Five percent of older adults are home bound, rarely leaving the home except for important medical appointments (59). Most of these older adults are >80. These older adults who live alone and are in poor health are vulnerable during a natural disaster (60).

Most older adults, even at advanced ages, live in the community. By 2035, the number of American households with someone over age 80 will double (61).

Approximately 13% of women and 8% of men over age 85 live in nursing facilities or other institutional settings (62). These rates have fallen in recent decades presumably due to less disability and better care options in the home. Support for home caregivers and promotion of home medical and social work services can further minimize institutionalization. More than 17 million Americans served as family caregivers to an older adult in 2011 (63). Being a caregiver is typically a prolonged responsibility although the number of hours of work involved markedly varies based on the needs of the care recipient. Older adults with dementia have the highest needs for caregiver time. Opportunities to support family caregivers can include formal training, peer counseling, stress management, legal advice, and employment-flexibility (64).

## Medical Decisions

Starting or stopping medications, ordering screening tests, sending people to the hospital, and advising families about placement or end of life care are complex discussions which health providers have with people over age 85. Often, family members play a central role in these complex discussions. The patient may have hearing impairment, cognitive impairment, or communication impairment. Life expectancy is often a major consideration; however, many patients are skeptical of life expectancy estimates and don’t like to talk about it (65).

### Polypharmacy

Polypharmacy is defined as the use of concomitant use of five or more medications by a single patient. When taking five medications, the risk of an adverse drug event or drug-drug interaction is very high. Polypharmacy increases the risk of falls, disability (66), and other negative outcomes. Providers must weigh time to benefit, burden, risks of adverse effects, and goals of care when choosing to start or stop medications in people over age 85 (67).

### Hospitalization

Hospitalizations are common among people over age 85 (68) and associated with functional decline (69). Providing more acute care in the home could help to prevent hospital complications such as functional decline and iatrogenic infection. Attention to transitional care and rapid post-hospital medical follow-up visits can minimize medication errors and rehospitalization.

### Institutional Placement

Many older adults value their ability to continue living in their own homes as they age. Home-based interventions may slow the progression of disability and prevent the need for institutionalization (70). Discussions with families review all of the options for living arrangements and then assess safety and preferences.

### Advance Directives and End of Life Care

Many 85-year olds with multiple chronic conditions will die within a few years. Advance directives on life-sustaining therapies such as cardiopulmonary resuscitation, mechanical ventilation, and tube feeding enable patients to exert some control over their end of life care. Every 85-year-old adult should appoint a health-care agent who can make complicated decisions in an emergency. As people approach the end of life, medical discussions tend to focus more on quality of life and symptom management. However, these “palliative” conversations are not only appropriate near the end of life. Clinicians should routinely assess symptoms and evaluate which problems affect a person’s quality of life.

## Conclusion

The aging process is universal but not uniform. Awareness of age-related physiological changes, such as reduced acuity of vision and hearing, slow reaction time, and impaired balance, will prepare patients and caregivers to manage risks, make informed decisions, and perhaps prevent falls and medication adverse effects.

Functional deterioration in an elderly person can also arise from social and mental health problems. Awareness of these problems may prevent age-related deterioration, such as attention to depression and suicide risk in men during the first year following the death of a spouse or depression after hip fracture or stroke.

Optimizing vision and hearing can prevent isolation, depression, and cognitive impairment. Lower extremity strength especially of the quadriceps muscle is critical for basic activities of daily living, especially bathing, walking, and performing transfers. People over age 85 need these muscles for stability and preventing falls. Walking speed is a helpful measure. Resistance exercise such as regular walking is recommended to help maintain strength and prevent cardiovascular disease. Maintaining a healthy body weight throughout the life span likewise can prevent diabetes, osteoarthritis, and other chronic diseases.

Decisions to prescribe medications or order screening tests should take into account goals of care, burden, risks, and lag time to benefit. In the future, more adults over age 85 will benefit from home-based services and technologies and will benefit from creative transportation and housing services opportunities for social participation, as well as programs to support family caregivers.

## Author Contributions

EJ and JB both contributed to the conception and writing of the paper.

## Conflict of Interest Statement

The authors declare that the research was conducted in the absence of any commercial or financial relationships that could be construed as a potential conflict of interest. The reviewer EC and handling editor declared their shared affiliation.

## References

[1] United Nations, Department of Economic and Social Affairs, Population Division. World Population Ageing 2015. (ST/ESA/SER.A/390). New York: United Nations (2015).

[2] Snider EL. Young-old versus old-old and the use of health services. Does the difference make a difference? J Am Geriatr Soc (1981) 29:354–8. 10.1111/j.1532-5415.1981.tb01241.x 7264125

[3] Gavazzi G Mallaret MR Couturier P Iffenecker A Franco A. Bloodstream infection: differences between young-old, old, and old-old patients. J Am Geriatr Soc (2002) 50:1667–73. 10.1046/j.1532-5415.2002.50458.x 12366620

[4] Suzman R Riley MW Introducing the “oldest old”. Milbank Mem Fund Q Health Soc (1985) 63:177–86. 10.2307/3349879 3846808

[5] Davis A McMahon CM Pichora-Fuller KM Russ S Lin F Olusanya BO Aging and hearing health: the life-course approach. Gerontologist (2016) 56(Suppl 2):S256–67. 10.1093/geront/gnw033 26994265 PMC6283365

[6] Desai M Pratt LA Lentzner H Robinson KN Trends in Vision and Hearing Among Older Americans. Aging Trends. Hyattsville, MD: National Center for Health Statistics (2001). 10.1037/e620682007-001 11894223

[7] Amieva H Ouvrard C Giulioli C Meillon C Rullier L Dartigues JF. Self-reported hearing loss, hearing aids, and cognitive decline in elderly adults: a 25-year study. J Am Geriatr Soc (2015) 63:2099–104. 10.1111/jgs.13649 26480972

[8] Popelka MM Cruickshanks KJ Wiley TL Tweed TS Klein BE Klein R. Low prevalence of hearing aid use among older adults with hearing loss: the epidemiology of hearing loss study. J Am Geriatr Soc (1998) 46:1075–8. 10.1111/j.1532-5415.1998.tb06643.x 9736098

[9] Evans JR Fletcher AE Wormald RP Ng ES Stirling S Smeeth L Prevalence of visual impairment in people aged 75 years and older in Britain: results from the MRC trial of assessment and management of older people in the community. Br J Ophthalmol (2002) 86:795–800. 10.1136/bjo.86.7.795 12084753 PMC1771210

[10] Zalewski CK. Aging of the human vestibular system. Semin Hear (2015) 36:175–96. 10.1055/s-0035-1555120 27516717 PMC4906308

[11] Dodds RM Granic A Davies K Kirkwood TB Jagger C Sayer AA. Prevalence and incidence of sarcopenia in the very old: findings from the Newcastle 85+ study. J Cachexia Sarcopenia Muscle (2017) 8:229–37. 10.1002/jcsm.12157 27897431 PMC5377385

[12] Walston JD. Sarcopenia in older adults. Curr Opin Rheumatol (2012) 24:623–7. 10.1097/BOR.0b013e328358d59b 22955023 PMC4066461

[13] Newman AB Kupelian V Visser M Simonsick EM Goodpaster BH Kritchevsky SB Strength, but not muscle mass, is associated with mortality in the health, aging and body composition study cohort. J Gerontol A Biol Sci Med Sci (2006) 61:72–7. 10.1093/gerona/61.1.72 16456196

[14] Xue QL Beamer BA Chaves PH Guralnik JM Fried LP Heterogeneity in rate of decline in grip, hip, and knee strength and the risk of all-cause mortality: the women’s health and aging study II. J Am Geriatr Soc (2010) 58:2076–84. 10.1111/j.1532-5415.2010.03154.x 21054287 PMC3058914

[15] Bandaranayake T Shaw AC. Host resistance and immune aging. Clin Geriatr Med (2016) 32:415–32. 10.1016/j.cger.2016.02.007 27394014 PMC6986475

[16] Raviotta JM Smith KJ DePasse J Brown ST Shim E Nowalk MP Cost-effectiveness and public health effect of influenza vaccine strategies for U.S. elderly adults. J Am Geriatr Soc (2016) 64:2126–31. 10.1111/jgs.14323 27709600 PMC5302117

[17] Gould L Abadir P Brem H Carter M Conner-Kerr T Davidson J Chronic wound repair and healing in older adults: current status and future research. J Am Geriatr Soc (2015) 63:427–38. 10.1111/jgs.13332 25753048 PMC4582412

[18] Ariathianto Y. Asymptomatic bacteriuria – prevalence in the elderly population. Aust Fam Physician (2011) 40:805–9. 22003486

[19] Zalmanovici Trestioreanu A Lador A Sauerbrun-Cutler MT Leibovici L. Antibiotics for asymptomatic bacteriuria. Cochrane Database Syst Rev (2015) 4:CD009534. 25851268 10.1002/14651858.CD009534.pub2 PMC8407041

[20] Collerton J Davies K Jagger C Kingston A Bond J Eccles MP Health and disease in 85 year olds: baseline findings from the Newcastle 85+ cohort study. BMJ (2009) 339:b4904. 10.1136/bmj.b4904 20028777 PMC2797051

[21] Wang JC Bennett M. Aging and atherosclerosis mechanisms, functional consequences, and potential therapeutics for cellular senescence. Circ Res (2012) 111:245–59. 10.1161/CIRCRESAHA.111.261388 22773427

[22] Alexander RW Hypertension and the pathogenesis of atherosclerosis. Hypertension (1995) 25:155–61. 10.1161/01.HYP.25.2.155 7843763

[23] Federal Interagency Forum on Aging-related Statistics. Older Americans 2016: Key Indicators of Well-Being. (2016). Available from: https://agingstats.gov/docs/LatestReport/Older-Americans-2016-Key-Indicators-of-WellBeing.pdf

[24] SPRINT Research Group Wright JT Jr Williamson JD Whelton PK Snyder JK Sink KM A randomized trial of intensive versus standard blood-pressure control. N Engl J Med (2015) 373:2103–16. 10.1056/NEJMoa1511939 26551272 PMC4689591

[25] Gorina Y Hoyert D Lentzner H Goulding M Trends in Causes of Death among Older Persons in the United States. Aging Trends. Hyattsville, MD: National Center for Health Statistics (2006). 19174841

[26] Siu AL U.S. Preventive Services Task Force. Screening for breast cancer: U.S. Preventive Services Task Force recommendation statement. Ann Intern Med (2016) 164:279–96. 10.7326/M15-2886 26757170

[27] Lee SJ Boscardin WJ Stijacic-Cenzer I Conell-Price J O’Brien S Walter LC. Time lag to benefit after screening for breast and colorectal cancer: meta-analysis of survival data from the United States, Sweden, United Kingdom, and Denmark. BMJ (2013) 346:e8441. 10.1136/bmj.e8441 23299842 PMC4688425

[28] Lin JS Piper MA Perdue LA Rutter C Webber EM O’Connor E Screening for colorectal cancer: updated evidence report and systematic review for the US Preventive Services Task Force. JAMA (2016) 315:2576–94. 10.1001/jama.2016.3332 27305422 PMC13623242

[29] Walter LC Covinsky KE. Cancer screening in elderly patients: a framework for individualized decision making. JAMA (2001) 285:2750–6. 10.1001/jama.285.21.2750 11386931

[30] Moyer VA U.S. Preventive Services Task Force. Screening for prostate cancer: U.S. Preventive Services Task Force recommendation statement. Ann Intern Med (2012) 157:120–34. 10.7326/0003-4819-157-2-201207170-00459 22801674

[31] Kirkman MS Briscoe VJ Clark N Florez H Haas LB Halter JB Diabetes in older adults. Diabetes Care (2012) 35:2650–64. 10.2337/dc12-1801 23100048 PMC3507610

[32] Odden MC Shlipak MG Whitson HE Katz R Kearney PM defilippi C Risk factors for cardiovascular disease across the spectrum of older age: the cardiovascular health study. Atherosclerosis (2014) 237:336–42. 10.1016/j.atherosclerosis.2014.09.012 25303772 PMC4254262

[33] Evidence Summary: Osteoporosis: Screening. U.S. Preventive Services Task Force (2014). Available from: https://www.uspreventiveservicestaskforce.org/Page/Document/evidence-summary6/osteoporosis-screening

[34] Ward BW Schiller JS. Prevalence of multiple chronic conditions among US adults: estimates from the National Health Interview Survey 2010. Prev Chronic Dis (2013) 10:E65. 10.5888/pcd10.120203 23618545 PMC3652717

[35] Hayek S Ifrah A Enav T Shohat T Presence correlates and time trends of multiple chronic conditions among Israeli adults: estimates from the Israeli National Health Interview Survey. Prev Chronic Dis (2017) 14:E64 10.5888/pcd14.170038 28796598 PMC5553352

[36] Gerteis J Izrael D Deitz D LeRoy L Ricciardi R Miller T Multiple Chronic Conditions Chartbook. Rockville, MD: Agency for Healthcare Research and Quality (2014). AHRQ Publications No, Q14-0038.

[37] Guralnik JM Ferrucci L Pieper CF Leveille SG Markides KS Ostir GV Lower extremity function and subsequent disability: consistency across studies, predictive models, and value of gait speed alone compared with the short physical performance battery. J Gerontol A Biol Sci Med Sci (2000) 55:M221–31. 10.1093/gerona/55.4.M221 10811152 PMC12149745

[38] Middleton A Fritz SL Lusardi M. Walking speed: the functional vital sign. J Aging Phys Act (2015) 23:314–22. 10.1123/japa.2013-0236 24812254 PMC4254896

[39] Butler AA Menant JC Tiedemann AC Lord SR. Age and gender differences in seven tests of functional mobility. J Neuroeng Rehabil (2009) 6:31. 10.1186/1743-0003-6-31 19642991 PMC2741473

[40] He W Larsen LJ U.S. Census Bureau, American Community Survey Reports, ACS-29, Older Americans with a Disability: 2008–2012. Washington, DC: U.S. Government Printing Office (2014).

[41] Jagger C Arthur AJ Spiers NA Clarke M. Patterns of onset of disability in activities of daily living with age. J Am Geriatr Soc (2001) 49:404–9. 10.1046/j.1532-5415.2001.49083.x 11347783

[42] Connolly D Garvey J McKee G. Factors associated with ADL/IADL disability in community dwelling older adults in the Irish longitudinal study on ageing (TILDA). Disabil Rehabil (2017) 39:809–16. 10.3109/09638288.2016.1161848 27045728

[43] WHO Global Report on Falls Prevention in Old Age. Geneva, Switzerland: World Health Organization (2007). Available from: http://apps.who.int/iris/handle/10665/43811

[44] Panel on Prevention of Falls in Older Persons, American Geriatrics Society and British Geriatrics Society. Summary of the Updated American Geriatrics Society/British Geriatrics Society clinical practice guideline for prevention of falls in older persons. J Am Geriatr Soc (2011) 59:148–57. 10.1111/j.1532-5415.2010.03234.x 21226685

[45] NICE. The Assessment and Prevention of Falls in Older People. (2013). Available from: http://www.nice.org.uk/CG161

[46] Fried LP Tangen CM Walston J Newman AB Hirsch C Gottdiener J Frailty in older adults: evidence for a phenotype. J Gerontol A Biol Sci Med Sci (2001) 56:M146–56. 10.1093/gerona/56.3.M146 11253156

[47] Bandeen-Roche K Seplaki CL Huang J Buta B Kalyani RR Varadhan R Frailty in older adults: a nationally representative profile in the United States. J Gerontol A Biol Sci Med Sci (2015) 70:1427–34. 10.1093/gerona/glv133 26297656 PMC4723664

[48] Puts MTE Toubasi S Andrew MK Ashe MC Ploeg J Atkinson E Interventions to prevent or reduce the level of frailty in community-dwelling older adults: a scoping review of the literature and international policies. Age Ageing (2017) 46:383–92. 10.1093/ageing/afw247 28064173 PMC5405756

[49] Shah D Badlani G. Treatment of overactive bladder and incontinence in the elderly. Rev Urol (2002) 4(Suppl 4):S38–43. 16986020 PMC1476020

[50] Sims J Browning C Lundgren-Lindquist B Kendig H. Urinary incontinence in a community sample of older adults: prevalence and impact on quality of life. Disabil Rehabil (2011) 33:1389–98. 10.3109/09638288.2010.532284 21692622

[51] Blazer DG Yaffe K Karlawish J Cognitive aging: a report from the Institute of Medicine. JAMA (2015) 313:2121–2. 10.1001/jama.2015.4380 25875498

[52] Rosen JB Brand M Kalbe E. Empathy mediates the effects of age and sex on altruistic moral decision making. Front Behav Neurosci (2016) 10:67. 10.3389/fnbeh.2016.00067 27147990 PMC4828431

[53] ADI. World Alzheimer Report 2015: The Global Impact of Dementia. London: Alzheimer’s Disease International (ADI) (2015).

[54] Amjad H Roth DL Samus QM Yasar S Wolff JL. Potentially unsafe activities and living conditions of older adults with dementia. J Am Geriatr Soc (2016) 64:1223–32. 10.1111/jgs.14164 27253366 PMC4914464

[55] Kenny RA Coen RF Frewen J Donoghue OA Cronin H Savva GM. Normative values of cognitive and physical function in older adults: findings from the Irish longitudinal study on ageing. J Am Geriatr Soc (2013) 61(Suppl 2):S279–90. 10.1111/jgs.12195 23662720

[56] Stek ML Vinkers DJ Gussekloo J van der Mast RC Beekman AT Westendorp RG. Natural history of depression in the oldest old: population-based prospective study. Br J Psychiatry (2006) 188:65–9. 10.1192/bjp.188.1.65 16388072

[57] Conwell Y Thompson C. Suicidal behavior in elders. Psychiatr Clin North Am (2008) 31:333–56. 10.1016/j.psc.2008.01.004 18439452 PMC2735830

[58] Steptoe A Shankar A Demakakos P Wardle J. Social isolation, loneliness, and all-cause mortality in older men and women. Proc Natl Acad Sci U S A (2013) 110:5797–801. 10.1073/pnas.1219686110 23530191 PMC3625264

[59] Ornstein KA Leff B Covinsky KE Ritchie CS Federman AD Roberts L Epidemiology of the homebound population in the United States. JAMA Intern Med (2015) 175:1180–6. 10.1001/jamainternmed.2015.1849 26010119 PMC4749137

[60] Dostal PJ. Vulnerability of urban homebound older adults in disasters: a survey of evacuation preparedness. Disaster Med Public Health Prep (2015) 9:301–6. 10.1017/dmp.2015.50 25906972

[61] Joint Center for Housing Studies of Harvard University. Projections and Implications for Housing a Growing Population: Older Households 2015-2035. Cambridge, MA: Harvard University (2016).

[62] Pew Research Center. Smaller Share of Women Ages 65 and Over Are Living Alone. Pew Research Center Social and Demographic Trends (2016). Available from: http://www.pewsocialtrends.org/2016/02/18/2-living-arrangements-of-older-americans-by-gender/

[63] National Academies of Sciences, Engineering, and Medicine. Families Caring for an Aging America. Washington, DC: The National Academies Press (2016). 27905704

[64] Carmeli E The invisibles: unpaid caregivers of the elderly. Front Public Health (2014) 2:91 10.3389/fpubh.2014.00091 25101257 PMC4104642

[65] Schoenborn NL Lee K Pollack CE Armacost K Dy SM Bridges JFP Older adults’ views and communication preferences about cancer screening cessation. JAMA Intern Med (2017) 177:1121–8. 10.1001/jamainternmed.2017.1778 28604917 PMC5564296

[66] Wang R Chen L Fan L Gao D Liang Z He J Incidence and effects of polypharmacy on clinical outcome among patients aged 80+: a five-year follow-up study. PLoS One (2015) 10:e0142123. 10.1371/journal.pone.0142123 26554710 PMC4640711

[67] Holmes HM Hayley DC Alexander GC Sachs GA Reconsidering medication appropriateness for patients late in life. Arch Intern Med (2006) 166:605–9. 10.1001/archinte.166.6.605 16567597

[68] Levant S Chari K DeFrances CJ Hospitalizations for Patients Aged 85 and over in the United States, 2000–2010. Hyattsville, MD: National Center for Health Statistics (2015). NCHS Data Brief No. 182. 25590465

[69] Boyd CM Xue QL Guralnik JM Fried LP. Hospitalization and development of dependence in activities of daily living in a cohort of disabled older women: the Women’s Health and Aging Study I. J Gerontol A Biol Sci Med Sci (2005) 60:888–93. 10.1093/gerona/60.7.888 16079213

[70] Szanton SL Wolff JL Leff B Roberts L Thorpe RJ Tanner EK Preliminary data from community aging in place, advancing better living for elders, a patient-directed, team-based intervention to improve physical function and decrease nursing home utilization: the first 100 individuals to complete a Centers for Medicare and Medicaid Services Innovation Project. J Am Geriatr Soc (2015) 63:371–4. 10.1111/jgs.13245 25644085 PMC4498670

